# The Diagnostic and Therapeutic Value of Micro-RNA in Parkinson’s Disease

**DOI:** 10.1007/s10571-026-01767-x

**Published:** 2026-06-26

**Authors:** Guo-Liang Lin, Yue Wang, Bi-Huan Cheng

**Affiliations:** Department of Critical Care Medicine, the Second Affiliated Hospital and Yuying Children’s Hospital, Wenzhou Medical University, Wenzhou, 325027 Zhejiang China

**Keywords:** Parkinson's disease (PD), α-synuclein (α-syn), Epigenetics, Micro-RNA (miRNA), Biomarkers

## Abstract

The pathogenesis of Parkinson’s disease (PD) remains incompletely understood, and effective early diagnostic biomarkers and disease-modifying therapies are lacking. MicroRNAs (miRNAs), a class of small non-coding RNAs, have emerged as critical regulators of gene expression involved in key pathological processes of PD, including neuronal apoptosis, mitochondrial dysfunction, oxidative stress, and α-synuclein (α-syn) aggregation. Recent evidence suggests that dysregulated miRNAs expression contributes significantly to PD progression and may serve as minimally invasive biomarkers for early diagnosis. Furthermore, miRNA-based therapeutic strategies offer promising avenues for targeting disease-related molecular pathways. This review summarizes the mechanistic roles of miRNAs in PD pathogenesis and highlights their potential clinical applications in diagnosis and therapy.

## Introduction

Parkinson’s disease (PD) is a common neurodegenerative disorder that primarily affects middle-aged and elderly people. The global prevalence is projected to increase from 6.2 million cases in 2015 to 19 million cases by 2040 (Dorsey and Bloem 2018). While the development of PD is associated with genetic susceptibility and chronic exposure to environmental toxins, the molecular mechanisms driving the disease remain enigmatic (Perinan et al. 2022). The Lewy body which is formed by abnormal aggregation of α-synuclein (α-syn) is considered the main pathological feature of PD (Blauwendraat et al. 2020, Gonzalez-Horta 2015, Dehay et al. 2015). Despite this, the potential pathogenetic molecular mechanisms of PD are not yet clear (Tryphena et al. 2023). Clinically, the diagnosis of PD is based on symptom recognition and radiological images (Blauwendraat et al. 2020). Researchers are increasingly recognizing the importance of early diagnosis of PD in delaying the progression of the disease (Doroszkiewicz et al. 2022). Unfortunately, current methods for early diagnosis of PD are remain limited, presenting a substantial challenge. Additionally, all pharmacologic and non-pharmacologic for PD patients are primarily symptomatic and have limited effects on disease progression (Armstrong and Okun 2020). Thus, exploring effective molecular tools to deeply understand the mechanisms underlying selective dopaminergic neuron loss in the substantia nigra may assist in early PD diagnosis and the identification of new treatment strategies to slow of PD progression.

MicroRNAs (miRNAs) are small non-coding RNA molecules, typically 21–25 nucleotides, that play a pivotal role in the regulation of gene expression. The discovery of miRNAs marked the inception of miRNA research, unveiling their pivotal role in nematode embryonic development and their influence on a diverse array of biological processes, including cell proliferation, differentiation, apoptosis, and neurological diseases (Lee et al. 1993, Gebert and MacRae 2019, Anglicheau et al. 2010, O’Brien et al. 2018, Saraiva et al. 2017). The biosynthesis of miRNAs encompasses a series of intricate steps and is widely studied as a potential therapeutic target (Zhang et al. 2023, Sadlon et al. 2019). Here is a simplified illustration of the miRNA maturation process:

DNA → pri-miRNA (Transcription by RNA polymerase II/III)

↓

(Drosha-DGCR8 complex cleavage) → pre-miRNA

↓

Exportin-5 (Nuclear export) → pre-miRNA (in cytoplasm)

↓

(Dicer cleavage) → dsRNA (22 nt)

↓

(Helicase activity) → Guide strand + Passenger strand

↓

Guide strand → RISC (RNA-induced silencing complex)

↓

RISC + target mRNA → Translational repression or mRNA cleavage

Here, we aim to review the regulatory mechanisms of miRNAs in neuronal apoptosis, mitophagy, oxidative stress, *SNCA* expression, neuroinflammation, as well as the potential value of miRNAs as biomarkers and therapeutic targets in the PD progression.

## miRNAs in PD Pathogenesis

### miRNAs Regulate Neuron Survival and Apoptosis in PD

Cell survival and death are important physiological processes within the body. They maintain homeostasis by regulating cell proliferation and clearance (Napoletano et al. 2019). However, mature neurons prossess no proliferatative and cannot be regenerated, making the study of neuronal death particularly important (Fricker et al. 2018). Various of extracellular or intracellular stresses can activate cell death cascades (Kayagaki et al. 2024). Abnormal cell death, including apoptosis, necrosis, pyroptosis, and ferroptosis can be observed in the pathogenesis of various neurodegeneration diseases (Moujalled et al. 2021).

**Fig. 1:**
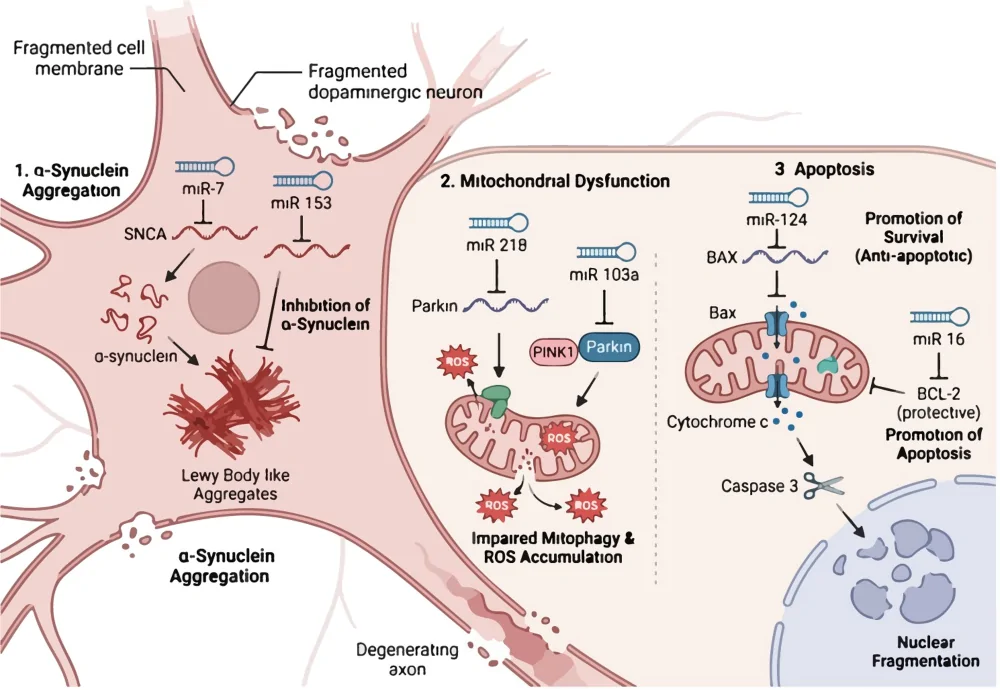
Mechanistic roles of miRNAs in Parkinson’s disease pathogenesis, including regulation of apoptosis, mitophagy, oxidative stress, and α-synuclein aggregation-. α-Synuclein pathology: miR-7 and miR-153 post-transcriptionally repress *SNCA* expression, thereby inhibiting α-synuclein aggregation and Lewy body formation. Mitochondrial dysfunction: The PINK1/Parkin pathway, which governs mitophagy, is negatively regulated by miR-218 and miR-103a, leading to the accumulation of damaged mitochondria and oxidative stress. Apoptotic cascade: miR-124 exerts neuroprotective effects by modulating the Bax/Bcl-2 ratio and inhibiting caspase-3 activation, thus preventing neuronal apoptosis. T-bars indicate inhibition; arrows indicate promotion or activation. Created in BioRender (https://biorender.com)

Apoptosis can be triggered through intrinsic (mitochondrial apoptosis) pathways and death receptor (also known as extrinsic) pathways. The trigger signal of apoptosis may include oxidative stress, DNA damage, and cytosolic Ca^2+^ overload (Dionisio et al. 2021). When cells receive apoptotic signals, pro-apoptotic proteins in the Bcl-2 family, such as Bax and Bad, elevate mitochondrial membrane permeability, triggering the release of apoptogenic factors, such as cytochrome c, from the mitochondrial interspace into the cytoplasm (Teresak et al. 2022, Flusberg and Sorger 2015). These factors activate the caspase cascade, primarily activating caspase-9, which then activates downstream caspases including caspase-3, caspase-6 and caspase-7. These caspases cleave various intracellular proteins, disrupting cell structure and function, and ultimately leading to programmed cell death (Dionisio et al. 2021). Conversely, the intrinsic anti-apoptotic mechanisms represent cellular protective response aimed at inhibiting or reversing the apoptosis. These mechanisms were summarized in the Fig. 1 and briefly described as follows:

1. Upregulating anti-apoptotic proteins, such as Bcl-2 and Bcl-xL, to suppress mitochondrial membrane permeability and prevent the release of apoptotic factors (Bock and Tait 2020).
2. Activation of survival signaling pathways, such as PI3K/Akt and MAPK/ERK, that promote cell survival and suppress apoptosis (Teresak et al. 2022).
3. Enhancing cellular antioxidant capacity to reduce oxidative damage. Subsequently, the promotion of DNA repair mechanisms to mend damaged DNA and avoid cells from entering the apoptotic process (Dionisio et al. 2021).

Intrinsic anti-apoptotic signalling shields cells from damage-induced death by maintaining the stability of the intracellular environment (Yuan and Ofengeim 2024). However, in the pathogenesis of many neurodegeneration diseases, this balance is disrupted, including PD (Vitale et al. 2023). Accordingly, the expression of caspase-3 and BAX in the brains of patients with PD is increased, whereas the Bcl-2 is decreased (Moujalled et al. 2021).

Extrinsic apoptosis is mediated by the activation of death receptors on the plasma membrane, whereas intrinsic apoptosis is triggered in the absence of death receptor signalling and is primarily regulated by mitochondrial pathways (Yuan and Ofengeim 2024). Although extrinsic apoptosis is a form of programmed cell death initiated by extracellular signals binding to cell surface death receptors, the relationship between extrinsic apoptosis pathways and PD remains unclear (Saadh et al. 2024).

MicroRNAs modulate the survival status of neurons by regulating the expression of Bcl-2 family members, such as miR-7 or miR-153. To date, both miRNAs are brain-enriched but are diminished in the substantia nigra of patients with PD (Ravanidis et al. 2020). Overexpression of miR-7 or miR-153 protects cortical neurons against 1-methyl-4-phenylpyridinium ion (MPP^+^, a neurotoxin)- induced toxicity, restored neuronal vitality and upregulate Bcl-2 levels, while reducing c-aspase-3 activation (Nies et al. 2021). miR-124 is one of the most abundantly expressed miRNAs in the brain, involved in neurogenesis, synaptic morphology, neurotransmission, inflammation, autophagy, and mitochondrial function. Studies have reported reduced miR-124 abundance in the substantia nigra of MPTP-induced PD model mice. Overexpression of miR-124 targets the BIM, a BH3-only protein involved in apoptosis protein, reduces intrinsic apoptosis, and ameliorates the loss of dopaminergic neurons caused by MPTP (Angelopoulou et al. 2019).

### miRNAs Modulate PD Pathology Through the Mitophagy Pathway 

Mitochondrial dysfunction and complex I deficiency have been linked to the loss of dopaminergic neurons and the development of PD (Fig. 1) (Malpartida et al. 2021, Gonzalez-Rodriguez et al. 2021). Typically, dopaminergic neurons demand substantial energy to sustain normal physiological functions. To satisfy this energy requirement, neurons depend on robust oxidative phosphorylation, a process that paradoxically generates reactive oxygen species (ROS). If the redox balance is not maintained, excess ROS can trigger neruonal oxidative stress and neurodegeneration (Wang et al. 2011). Disruption of the delicate balance between energy supply and demand triggers degeneration of dopaminergic neurons in pathological conditions, such as dysfunctional in PTEN-induced putative kinase 1 (PINK1) and Parkin (Malpartida et al. 2021). Furthermore, the cytotoxic induced by misfolded proteins may be one of the possible reasons for selective neuronal degeneration. Such abnormal protein folding can be recognized and cleared by cellular systems such as autophagy-lysosome and ubiquitin-proteasome pathways. Mitophagy, as a specific form of autophagy, removes damaged mitochondria and maintains cellular homeostasis (Eldeeb et al. 2022). PINK1/Parkin-mediated ubiquitin-mitophagy is a well-characterized autophagic pathway (Li et al. 2023). In intact mitochondria, PINK1 is located in the outer membrane of mitochondria and is quickly cleaved (Wang et al. 2011). When mitochondria are damaged, PINK1 recruits and phosphorylates Parkin, initiating a cascade that leads to the ubiquitination, forming polyubiquitin chains (Malpartida et al. 2021). These phospho-ubiquitin chains recruit autophagy receptors, such as SQSTM1/p62 and OPTN, further promoting the docking between damaged mitochondria and autophagic membranes (Vargas et al. 2023). Such receptors mark damaged mitochondria as targets for the formation of autophagosomes, which then transport the damaged mitochondria to lysosomes for degradation (Nakatogawa 2020).

**Fig. 2:**
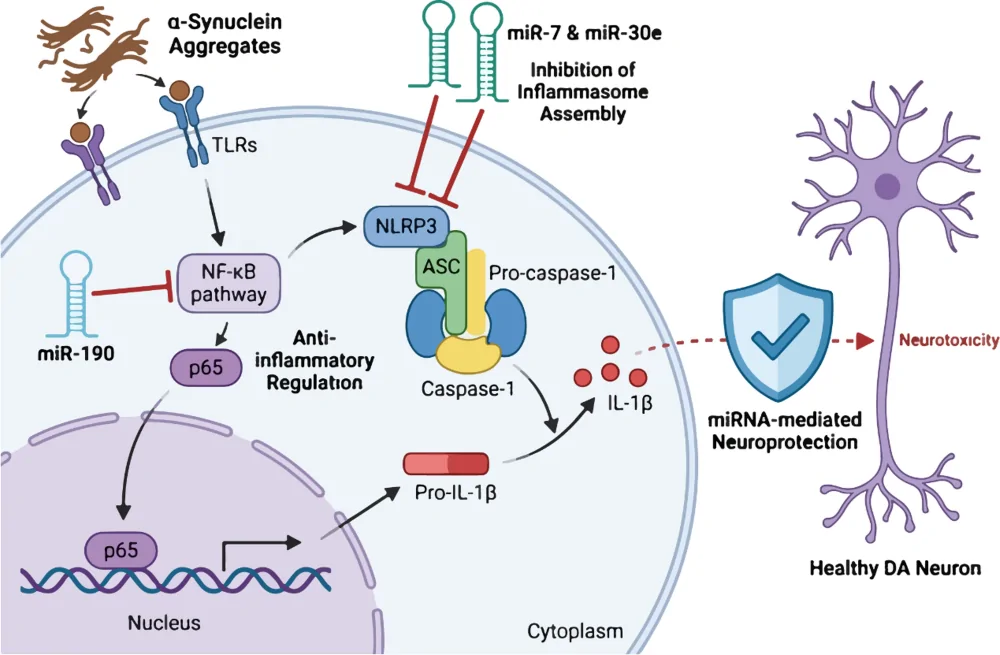
The assembly and effector functions of the NLRP3 inflammasome in the context of PD-associated neuroinflammation. Upon activation by danger signals (e.g., misfolded α-synuclein), NLRP3 recruits the adaptor protein ASC and pro-caspase-1 to form the inflammasome complex. This assembly facilitates the autocatalytic cleavage of pro-caspase-1 into active caspase-1, which matures proinflammatory cytokines pro-IL-1β and pro-IL-18 into secreted bioactiveIL-1β and IL-18. The diagram highlights the protective roles of miR-7, miR-30e, and miR-190, which attenuate neuroinflammation by directly targeting NLRP3 components or blocking upstream NF-κB signaling. Created in BioRender (https://biorender.com)

Recent studies have suggested the dysfunctional PINK1/Parkin mitophagy pathway in neurodegeneration and familial PD (Jankovic and Tan 2020, Bloem et al. 2021). Yet, the interplay between miRNAs and the PINK1/Parkin complex in the progression of PD remains not fully elucidated. Gomes et al. analyzed 19 PD patients and 12 controls from the Parkinson’s UK Brain Bank andidentified several downregulated PD-associated genes, including miR-218-5p (Caldi Gomes et al. 2022). miR-218 targets Parkin mRNA, thereby diminishing Parkin content and disrupting the function of the E3 ubiquitin ligase. This defect reduces mitochondrial ubiquitination and compromises autophagic clearance of damaged mitochondria (Rita et al. 2020). Despite this, the authors did not address the differences in PINK1-Parkin-mediated mitophagy between in-vitro and in-vivo models (Han et al. 2023). Nonetheless, their findings confirmed that miR-218 regulates Parkin expression and mitophagy. miR-103a-3p, which is highly expressed in the human brain, was found to be significantly increased in 1-methyl-4-phenyl-1,2,3,6-tetrahydropyridine (MPTP)-treated mice and MPP^+^ treated SH-SY5Y cells, with a concomitant significant reduction in Parkin expression. Functional experiments demonstrate that miR-103a-3p overexpression disrupts mitophagy by inhibiting Parkin-mediated mitophagy, while the inhibition of miR-103a-3p plays a neuroprotective role and enhances mitophagy, an effect that is strongly negated by Parkin siRNA. These findings suggest that the inhibition of miR-103a-3p may offer neuroprotection in PD by modulating mitophagy through the Parkin pathway (Zhou et al. 2020). Similarly, miR-593-5p and miR-27a/b inhibit PINK1 transcription (Yoo et al. 2023, Kim et al. 2016), modulating the PINK1/Parkin signaling pathway and leading to cell death (Yoo et al. 2023). miR-27a/b strongly downregulate PINK1 expression, resulting in the failure of Parkin to translocate to mitochondria, diminished ubiquitin phosphorylation, reduced accumulation of LC3-II, and ultimately, impaired mitophagy (Kim et al. 2016). Collectively, these studies indicate that certain miRNAs can interfere with the mitophagy process, diminishing the cellular capacity to clear toxic substances and potentially impacting neuronal survival in PD (Table 1).

**Table 1:**
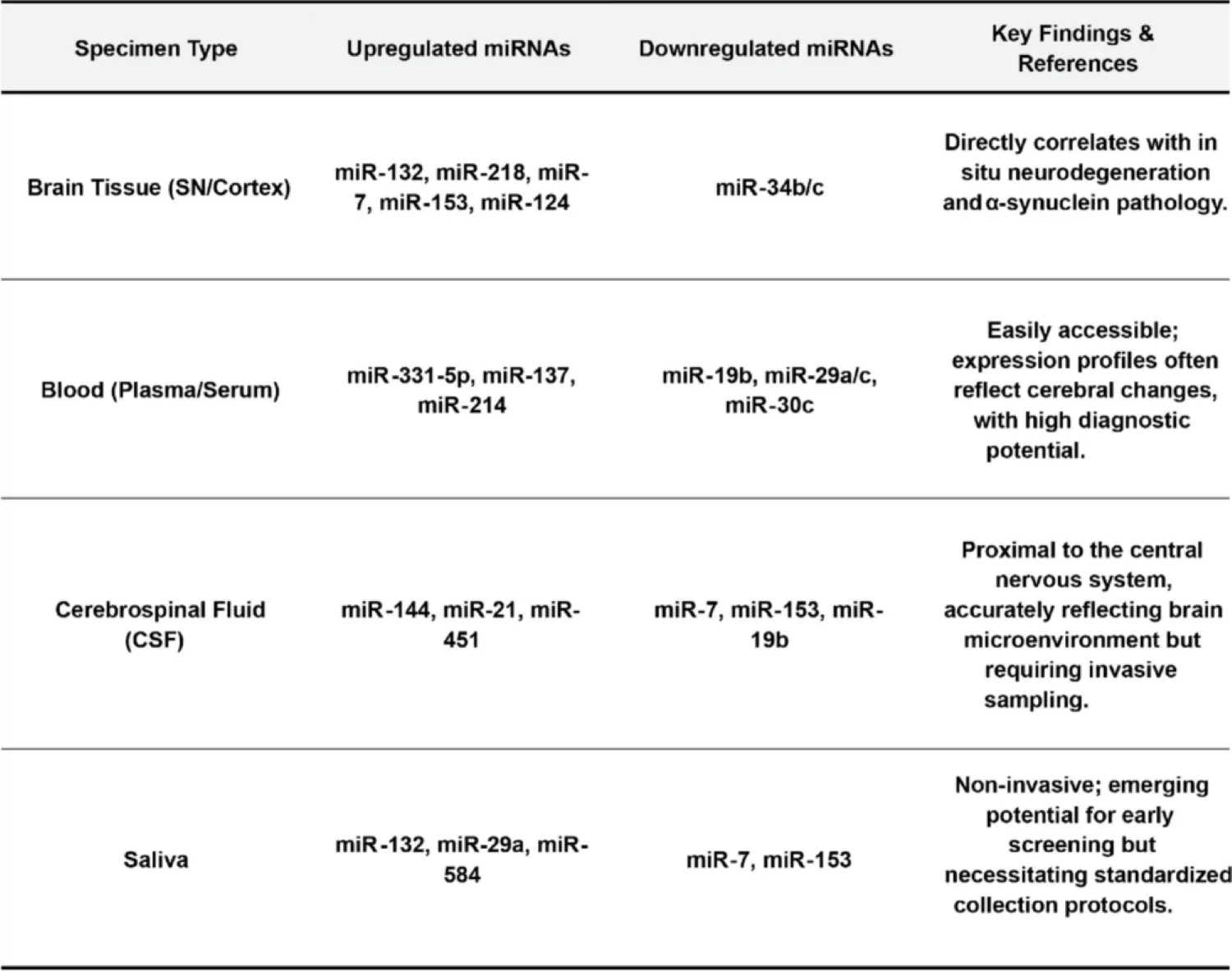
The candidate miRNA biomarkers reported in different sample types along with their diagnostic features

miRNAs derived from cerebrospinal fluid can directly reflect pathological changes in the central nervous system and have high diagnostic specificity, but sample acquisition is highly invasive; Blood (serum/plasma) samples are easy to obtain and have strong stability, making them suitable for routine screening; Saliva samples have the lowest invasiveness and are convenient for home testing; miRNAs derived from extracellular vesicles can avoid degradation and are enriched in molecules derived from neurons, possessing both stability and pathological specificity. The miRNA biomarkers of various sample types exhibit different trade-off characteristics between diagnostic convenience and pathological correlation.

### miRNAs and the Regulation of Oxidative Stress Responses

ROS production mainly comes from dysfunctional mitochondria. Mitochondrial dysfunction triggers disrupted energy metabolism and impaired oxidative phosphorylation, representing a potential mechanism underlying PD pathogenesis (Fig. 1) (Malpartida et al. 2021). This disruption culminates in the excessive generation of ROS, which can be triggered by various etiologies, including extrinsic and intrinsic sources. In PD, extrinsic sources of ROS involve chronic exposure to environmental toxins such as organochlorines, heavy metals, paraquat, and MPTP (Dionisio et al. 2021).

MPTP, recognized for its ability to induce PD-like symptoms in experimental animals (Meredith and Rademacher 2011), operates through a cascade of pathogenic mechanisms that encompass mitochondrial damage, oxidative stress, the incitement of immunological inflammatory responses and the cell death (Henrich et al. 2023, Vellingiri et al. 2022, Dauer and Przedborski 2003). As a lipophilic molecule, MPTP readily crosses the blood-brain barrier and, within the brain, undergoes biotransformation by astrocytic monoamine oxidase B (MAO-B) into the intermediate metabolite, 1-methyl-4-phenyl-2,3-dihydropyridine (MPDP^+^) (Meredith and Rademacher 2011). Subsequently, MPDP^+^ undergoes self-oxidation to form the active neurotoxin 1-methyl-4-phenylpyridinium (MPP^+^). MPP^+^ is selectively internalized by dopaminergic neurons through dopamine transporters (DAT), leading to its accumulation within these cells (Langston 2017, Huenchuguala and Segura-Aguilar 2024). This accumulation subsequently inhibits complex I of the respiratory chain, curtailing ATP synthesis and augmenting ROS production, particularly the generation of superoxide anions (Hu and Wang 2016). Besides the aforementioned factors, research indicates that ROS also originates from NADPH oxidase and flavoproteins, all of which may facilitate PD initiation. (Jomova et al. 2023).

Furthermore, the metabolism of dopamine, particularly its oxidation in the cytoplasm or extracellular space, especially under neutral to alkaline pH conditions, produces ROS, contributing to oxidative stress in PD (Bhattacharjee and Borah 2016, Graves et al. 2020, Watanabe et al. 2024). Dopamine semiquinone free radicals formed during this process can engage in Fenton reactions, interacting with iron ions (Fe2+) to generate highly reactive hydroxyl radicals (⋅OH). Together with superoxide anions (O2⋅−) produced in the Haber-Weiss reaction, these radicals exacerbate oxidative stress, leading to lipid peroxidation and cellular damage (Schildknecht et al. 2017). The metabolite 6-hydroxydopamine (6-OHDA), a neurotoxin, further participates in oxidative reactions, perpetuating ROS production.

Generated ROS instigate a cascade of reactions that inflict extensive oxidative damage to cellular components, including proteins, lipids, and DNA (Jomova et al. 2023, Pfeifer 2024). Persistent oxidative stress perturbs the intracellular milieu, precipitating cell death pathways such as apoptosis and necroptosis (Dionisio et al. 2021). Moreover, oxidative stress activates microglia, instigating neuroinflammation and exacerbating neurodegeneration. It may also promote the misfolding and aggregation of α-synuclein (α-syn) (Masato et al. 2019). At the same time, the aggregation of α-syn and neuroinflammation can feed back oxidative stress in neurons (Jomova et al. 2023, Tansey et al. 2022). This ROS-driven loop further impairs mitochondrial function, suppresses ATP synthesis, and establishes a self-perpetuating vicious cycle of oxidative damage (Dauer and Przedborski 2003). Evidence has demonstrated that miRNAs regulate cellular redox balance by reducing free radical production or enhancing antioxidant enzyme activity. As described above, miR-103a-3p, miR-218, and miR-27a/b act as regulators PINK-Parkin-mediated mitophagy and mitochondrial function. Downregulation of miR-7 contribute to α-syn aggregation, activates neuroinflammation, and lead to increase ROS levels (Zhang et al. 2021). miR-124 decreased NOX1 (an NADPH oxidase, responsible for reactive oxygen species generation) expression and depress the α-syn accumulation in the substantia nigra of paraquat-lesioned PD rats (Esteves et al. 2024). The overexpression experiments found that miR-137 regulate the DAT expression and reduce the dopamine uptake (Jia et al. 2016). However, the quantity of miR-137 was found to be unaltered in post-mortem substantia nigra samples of PD patients (Cardo et al. 2014, Bodai et al. 2024).

### miRNAs in the Regulation of *SNCA* Expression

Accumulatin research has underscored the pivotal role of aberrant gene expression in PD, with a particular focus on the expression of the *SNCA* (Morris et al. 2024). Recent advanced research has identified *SNCA* or its expressed product, α-syn, as a therapeutic target for PD, providing a promising pathway for treatment (Dehay et al. 2015, Grosso Jasutkar et al. 2022). α-syn-driven molecular pathogenesis in PD is involved in several key processes, including the ubiquitin–proteasomal system, autophagy–lysosomal pathway, mitochondrial maintenance and integrity, oxidative stress, and neuroinflammation (Li et al. 2020). Therefore, uncovering the regulatory mechanisms governing *SNCA* expression is essential for comprehending the developmental process of PD. Evidence indicates that the supports a link between variable 3'UTR lengths and their modulation of *SNCA* expression and PD pathogenesis (Mouradian 2012). miR-7 and miR-153 have been identified as regulators of α-syn expression by binding to the 3’UTR of *SNCA* (Fig. 1). The reduction of these miRNAs is associated with enhanced α-syn production in primary neurons (Mouradian 2012, Doxakis 2010). Moreover, the overexpression of miR-7 by stereotaxic injection of AAV-miR-7 followed by inoculation with α-syn pre-formed fibrils (PFF) results in a reduction of α-syn expression in the striatum and substantia nigra, accompanied by less dopaminergic neurodegeneration, and improves behavioral defects in mice (Zhang et al. 2021). Furthermore, in PD mice, downregulation of miR-26a has been found to increase the expression of death-associated protein kinase 1 (DAPK1) by post-transcriptional regulation, resulting in α-syn accumulation and neurodegeneration (Su et al. 2019).

However, several studies have uncovered that miRNA levels in the plasma of PD patients are elevated, but their expression in the midbrain tends to decrease, as observed with miR-7 and miR-433 (Ravanidis et al. 2020, Yang et al. 2021). Concurrently, plasma α-syn levels are increased in PD patients and show a positive correlating with UPDRS-III motor scores (Fan et al. 2020). This discrepancy suggests that additional, as yet unidentified, pathways may be at play in the regulation of *SNCA* gene expression, warranting further exploration and research.

### miRNAs Regulate NLRP3 Inflammasome Activation in PD

Recent findings have highlighted neuroinflammatory mechanisms in the onset and progression of PD (Tansey et al. 2022). Activated microglia and the release of chronic inflammatory factors mediate the loss of dopaminergic neurons in post-mortem PD brains and animal models of PD (Wang et al. 2015). In the early stages of the disease, environmental toxins such as MPTP and 6-hydroxydopamine (6-OHDA) damage dopaminergic neurons in the substantia nigra, leading to impaired α-syn clearance, activation of microglia, and the release of pro-inflammatory factors such as interleukin-1β (IL-1β), interleukin-18 (IL-18), and tumor necrosis factor-α (TNF-α). These pro-inflammatory factors further activate inflammasomes, stimulate and exacerbate dopaminergic neuronal damage, leading to dopaminergic neurodegeneration (Haque et al. 2020). The NLRP3 inflammasome, a multi-protein complex central to this inflammatory response is described in Fig. 2. The activation of the NLRP3 inflammasome in PD is a complex process involving various intracellular signals and molecular mechanisms (Han and Le 2023, Voet et al. 2019, Zhang et al. 2020, Huang et al. 2021), which can be summarized in Fig. 2.

In our current understanding, microglial NLRP3 inflammasome activation plays a critical pathogenic role in PD. NLRP3 deficiency significantly reduces motor dysfunctions, suppresses microglial recruitment, diminishes IL-1β production and inhibits caspase-1 activation. These effects collectively contribute to an amelioration of dopaminergic neurodegeneration of MPTP-treated mice (Lee et al. 2019). In addition, studies have suggested that miRNAs fine-tuning the activation of the NLRP3 inflammasome in PD (Han and Le 2023). For instance, miR-7 directly bindto NLRP3 mRNA, significantly inhibiting its expression. This reduces NLRP3 inflammasome activation, blocks the subsequent cascade inflammatory response, and alleviating the loss of dopaminergic neurons (Zhou et al. 2016). The upregulation of miR-30e and miR-190 results in reduced expression of TNF-α, COX-2, and iNOS, attenuating the loss of tyrosine hydroxylase in the substantia nigra (Li et al. 2018, Sun et al. 2019). Besides, researchers discovered that miR-30 agomir suppresses α-syn expression in the substantia nigra and ameliorates behavioral deficits in MPTP-mouse models (Li et al. 2018). miR-124 exhibits downexpression in the PD patients’ midbrain tissues compared to the healthy controls (Kim et al. 2007). miR-124 overexpression reduced the microglia activation, attenuated NF-κB signaling, and promotes autophagy in the inflammatory pathogenesis in the substantia nigra par compacta of MPTP-treated mice (Yao et al. 2018, Yao et al. 2019). However, there are reports showing that other examples of miRNAs upregulate neuroinflammation and contribute to PD pathogenesis, such as miR-155. miR-155 deletion dampens proinflammatory responses triggered by α-syn and blocked α-syn-induced neurodegeneration (Thome et al. 2016).

### miRNAs as Potential Diagnostic Biomarkers for PD

The scientific community has posited that aberrant miRNAs may play a role in the regulation of genes associated with PD, with their dysregulation potentially contributing to the onset and progression of the disease. Kim et al. examined the expression of 224 miRNAs in post-mortem brain samples from PD patients and healthy controls. The study revealed that eight miRNA precursors (pre-let7a-1, pre-miR-7-2, pre-miR-99a, pre-miR-130, pre-miR-133b, pre-miR-136, pre-miR-224, and pre-miR-143) were more than two-fold enriched in the midbrain of PD patients (Kim et al. 2007). Notably, miR-133b is enriched in the midbrain of both PD patients and normal mice but exhibited heterogeneity in the 6-OHDA PD model. Sébastien commented that the small sample size (3 PD patients and 5 controls) and high sample heterogeneity lead to the results arguable (Hebert and Strooper 2007). Insufficient tissue separation and heterogeneity of subcellular structure may explain the inconsistent expression profiles observed between the 6-OHDA PD model and PD patients and normal mice datasets. Nelson and Wilfred suggested that in situ hybridization is an essential experimental technique to complement miRNA expression profiling, providing a more nuanced understanding of miRNA distribution and function (Nelson and Wilfred 2009). In another study, Asikainen and his colleagues discovered that overexpressing the human A53T mutant α-syn in the nematode *Caenorhabditis elegans* led to the modulation of specific miRNAs (miR-64 and miR-65) via PD-associated genes (mdl-1 and ptc-1) (Asikainen et al. 2010). This finding prompts a rethink of whether the aberrant expression of miRNAs in the midbrain is a result of their dysregulated control in surviving neurons or merely a consequence of dopaminergic neuronal degeneration (Mouradian 2012).

However, miRNAs in the biofluids, such as saliva, cerebrospinal fluid (CSF), and plasma, which are termed circulating miRNAs, are easier to obtain and less-invasive than brain tissue biopsies for early PD diagnosis (Kuo et al. 2021). Kern et al. have adopted a minimally invasive approach to detect early signs of PD by employing RNA sequencing technology to scrutinize small non-coding RNAs in 5,450 blood samples from 1,614 individuals. Their investigation uncovered that, in total PD cases (both idiopathic and genetic), three miRNAs (miR-487b-3p, miR-493-5p, and miR-15b-5p) were significantly downregulated, while two miRNAs (miR-6836-3p and miR-6777-3p) exhibited upregulation compared with healthy controls. The study further examined the correlations between miRNA expression and disease progression, revealing that over 70 miRNAs displayed aberrant expression in patients with advancing PD, as opposed to those with stable disease progression. Cluster analysis indicated a downregulation of miRNAs, particularly in lymphocytes, with concurrent upregulation observed in serum and neutrophils. The candidat miRNAs converge on inflammatory and mitochondrial pathways, which are key in the pathology of PD. It is noteworthy that the molecular onset age of PD has two peaks, occurring in the late thirties and late sixties, suggesting that the dysregulation of miRNA expression is significantly age-dependent. However, the findings only partially align with the validation data from the Luxembourg Parkinson’s Study, a sub-cohort of the National Centre for Excellence in Research on PD (NCER-PD). Methodological disparities and variability in disease stages are critical factors that must be taken into account when interpreting these results (Kern et al. 2021).

Saliva offers a more convenient and non-invasive alternative to blood for sample collection. In the quest for diagnostic biomarkers for PD, researchers have primarily focused on salivary biomarkers such as α-syn, DJ-1 protein, and tumor necrosis factor-α (TNF-α) (Bartolo et al. 2024). However, the potential of salivary miRNAs as diagnostic aids for PD has been less explored. A recent study reported significantly lower expression of miR-153 and miR-223 in the saliva of PD patients compared to healthy controls, whereas miR-7 exhibited no comparable alteration. The study indicated that miR-153 had a sensitivity of 81.8% and a specificity of 71.4%, while miR-223 reached a sensitivity of 72.7%. The authors correlated these miRNA changes with clinical progression, using miR-153 and miR-223 as candidate biomarkers. Following a 2.78 years clinical follow-up, significant differences from controls were observed only in patients at Hoehn and Yahr stage 2. As biomarkers, these miRNAs’ diagnostic potential is complicated by overlaps with criteria for other neurological diseases, including Alzheimer’s disease (Cressatti et al. 2020). A study from China further investigated the expression of miRNAs in saliva samples from PD patients and healthy controls, aiming to evaluate their potential as PD biomarkers. The team identified two miRNAs, miR-29a-3p and miR-29c-3p, significantly downregulated in PD patients, with sensitivities of 79.3 and 65.4%, and specificities of 51.2% and 70.6%, respectively. By contrast,, miR-6756-5p was significantly upregulated in PD patients, with a sensitivity of 66.7% and a specificity of 58.6%. The combined use of miR-29a-3p and miR-29c-3p improved diagnostic sensitivity and specificity to 66.7% and 83.8%, respectively, with an area under the ROC curve (AUC) of 0.773, suggesting that this miRNA combination enhances the diagnostic accuracy for PD. Notably, miR-29a-3p showed high specificity in distinguishing PD from multiple system atrophy (MSA), with an AUC of 0.894, a sensitivity of 65.0%, and a specificity of 90.0% (Jiang et al. 2021). While the use of salivary miRNAs as biomarkers has its limitations, the application of miRNA panels in body fluids, including serum and saliva, holds promise as potential biomarkers for the diagnosis of PD. The pursuit of such non-invasive diagnostic tools is crucial for early and accurate detection of PD, facilitating better patient management and therapeutic intervention (Li et al. 2020).

miRNAs in CSF have garnered considerable interest as potential biomarkers for PD. A study by Starhof et al. was conducted through a two-step exploratory study design, first screening 372 miRNAs in a small-scale pilot cohort (*n* = 40), and then validating these findings in an independent study cohort (CSF *n* = 118 and plasma EDTA plasma *n* = 114). The research results indicate that specific combinations of CSF miRNAs exhibit high diagnostic accuracy in differentiating PD, MSA, and progressive supranuclear palsy (PSP) from a healthy controls. In particular, the combination of miR-7-5p, miR-331-5p, and miR-145-5p demonstrated an AUC of 0.88 for distinguishing PD from the controls. Similarly, the combination of miR-7-5p, miR-34c-3p, and miR-let-7b-5p achieved an AUC of 0.87 when differentiating MSA from the controls. Moreover, the individual miRNA miR-106b-5p showed the greatest discrimination ability between PD and PSP, with an AUC of 0.85.

These findings suggest that miRNAs in CSF may serve as biomarkers for PD, helping to improve diagnostic accuracy and early recognition of diseases. However, Starhof et al. pointed out that the expression levels of certain miRNAs (miR-7-5p and miR-204-5p) in PD have undergone significant changes; these molecules showed no significant correlation with CSF α-syn concentrations, indicating that miRNAs contribute to PDpathogenesis through alterative regulatory mechanisms.

In addition, the authors also investigated the differential expression of miRNAs in the CSF and plasma of PD and MSA with Parkinson’s syndrome. The study found significant differences in the expression of miRNAs in CSF and plasma of PD and MSA. Such divergent miRNA expression may be influenced by various factors, including sample collection and processing methods, detection techniques used, disease stages, and individual differences. Therefore, these results require validation in larger independent cohorts and further studied to determine their specific roles in disease development and biomarkers potential (Starhof et al. 2019).

### miRNAs in PD Therapy

The treatment for PD mainly includes drug therapy and non-drug therapy (Foltynie et al. 2024). Pharmacological treatments includes traditional dopamine like drugs and cholinergic receptor blockers, while new drugs include small molecule drugs, calcium channel blockers, iron scavengers, anti-inflammatory therapy, and immunotherapy (Elkouzi et al. 2019). Many traditional drug treatments have limitations in that they are only used to improve motor symptoms, but have no significant effect on disease outcomes (Hill et al. 2023). At the same time, the differences in diseases among different patients are the difficulty of drug treatment. Therefore, researchers hope to use new drugs to alleviate PD symptoms, delay disease progression and improving prognosis. In the past decades, studies have focus on biologic therapy for PD, including gene therapy, cell replacement therapy (Deverman et al. 2018), and immunotherapy (Pagano et al. 2024). In the past few decades, significant progress has been made in the field of gene therapy in treating genetic diseases that were previously untreatable (Cring and Sheffield 2022). While it is estimated that more than 60% genes are regulated by miRNAs (Saadh et al. 2024, Sayed and Abdellatif 2011). In this section, we discuss how miRNAs regulate the expression of key genes involved in PD, and how they influence motor symptoms and disease progression through various drug delivery strategies.

### Adeno-Associated Viral Vector Carrying miRNAs as Gene Therapy Platforms for PD 

In the past decades, adeno-associated viral (AAV) packaging modified drug delivery systems have been widely used in research on neurological diseases, including Alzheimer’s disease, prefrontal dementia, and Parkinson’s disease. Transgenes encoding therapeutic miRNAs have been successfully delivered to the central nervous system (CNS) with AAVs in mice and other species (Deverman et al. 2018). miR-7 can downregulated the expression of α-syn by combining 3’UTR within *SNCA* (Doxakis 2010). After injecting AAV-miR-7 into the dorsal striatum of mice, researchers found that miR-7 can protect dopaminergic neurons in the substantia nigra. Compared with the control group, miR-7 can alleviate motor dysfunction caused by PFF (Zhang et al. 2021). miR-181a is abundant in the brain and increases in brain samples of Parkinson’s disease patients. miR-181 targeted mRNA is widely downregulated in aging and Parkinson’s disease brains. Researchers designed AAV-miR-181a/b to overexpress miR-181a/b, and found an increase in α-syn induced dopamine neuron loss, while miR-181 inhibitors have neuroprotective effects (Stein et al. 2022).

However, there are still some issues with miRNA therapeutic strategies, such as safety and dosing. Studies have reported severe liver dysfunction and degeneration of dorsal root ganglia sensory neurons caused by AAVhu68, an AAV9 variant (Hinderer et al. 2018). Another adverse event was reported three months after stereotactic injection of rAAV2/1-miS1 in non-human primates' brain. The experimental animals developed ataxia, tremor, head-tilt and dysmetria (Keiser et al. 2021).

Another problem is so called "off-target"effects,which can hardly be avoided because endogenous miRNAs administered in therapies often exceed the physiological expression range of endogenous miRNAS (Diener et al. 2022). Off-target effects denote unintended binding and regulatory interaction between miRNAs and non-target mRNAs, potentially resulting in unforeseen biological outcomes (Zhang et al. 2021). miRNAs generally bind to the 3'UTR of target mRNAs (He and Hannon 2004), though researchers have discovered that partial complementarity can lead to non-target interactions (Segal and Slack 2020). Additionally, excessive extrinsic miRNA may heighten the probability of binding to non-targets. Therefore, researchers suggest reducing the dosage to mitigate the risk of off-target effects (Lai et al. 2019). Furthermore, strategies to minimize off-target effects include careful selection of miRNA sequences with low potential for cross-hybridization with non-target genes, the use of chemically modified miRNAs to improve specificity, and the development of algorithms to predict and filter out potential off-target interactions (Diener et al. 2022). In neurodegenerative disease treatments, off-target effects could result in several issues, such as decreased therapeutic efficacy, increased side effects (Jackson et al. 2003). In some cases, treatments may broadly target multiple pathways impacting neurodegenerative diseases (Farh et al. 2005). However, this approach also increases the likelihood of off-target effects. Furthermore, the long-term toxicities induced by off-target effects in the brain are not always immediately evident and may become apparent only with prolonged treatment (Jackson and Linsley 2010).

As of now, 6 registered recombinant AAV(rAAV) related gene therapy products and 87 ongoing clinical trial proofs have been reported (Slyk et al. 2024). The rAAV vectors, include rAAV2, rAAV9 and AAVrh10 are widely used in the CNS in several clinical trial. Although the etiology of dopaminergic neuron loss in PD is not yet clear, several gene therapy strategies have been clinically evaluated. These studies focus on four main targeting methods, namely (1) restoring dopamine synthesis; (2) Neuroprotection; (3) Genetic neural regulation; (4) Regulation of disease modifying variants (Ng et al. 2023). Recently, rAAV2-GAD (glutamic acid decarboxylase, Identifier: NCT00643890) and rAAV2-AADC (aromatic L-amino acid decarboxylase, an enzymes related to dopamine synthesis, Identifier: NCT02418598) was approved by the US Food and Drug Administration (FDA) for phase I/II clinical trials. Three distinct dose cohorts were set for AAV2-GAD with concentration of: 1 × 10^11^ vg/mL, 3 × 10^11^ vg/mL, and 1 × 10^12^ vg/mL, all delived via stereotactic injected into the STN of PD patients. The Phase I trial included 12 patients who received a unilateral infusion of 50 µ L AAV2-GAD to STN in the hemisphere with more severe symptoms. Perform UPDRS scoring 12 months after infusion. In the phase II trial, 16 subjects received bilateral AAV2-GAD infusion (1 × 10^12^ vg/mL), with clinical outcome evaluations completed at 6 months later. Two stages of clinical research have observed improvements in patients’ motor symptoms (Mendell et al. 2021). However, in a clinical study of AAV2-hAADC delivery to the putamen, participants developed movement disorders that recurred one year after treatment discontinuation. Real time monitoring of the treated volunteers’ head magnetic resonance imaging revealed that gradually increased bilateral putamen asymptomatic hemorrhage, thus terminating clinical observation (Ng et al. 2023). To date, no AAV vector carrying microRNA for PD therapy have progressed to clinical-stage testing. But we believe that with the development of engineering and manufacturing technologies, AAV as a gene therapy strategy will be more perfect.

### Exosome- and Nanotechnology-Based Drug Delivery Systems: Emerging Frontiers in PD Management

Aside from viral systems for drug delivery research, exosomes have attracted extensive research attention in recent years (Haney et al. 2015). As cell-derived, with a diameter of approximately 50–150 nanometers, exosomes serving as carriers for information transmission between adjacent and distal cells and mediate intercellular communication (Kojima et al. 2018). On the one hand, evidence has shown α-syn spread from neuron to neuron and exosomes are considered as the important mediator (Calabresi et al. 2023). Additionally, the extracellular α-syn could transmitted by exosomes from neuron to immunocyte, inducing astrocyte and microcyte neuroinflammatory response (Izco etal. 2022). On the other hand, extracellular trafficking of miRNAs constitutes a critical mode of intercellular signal transduction, which greatly increases their biological functional impotance by reshaping the genetic profiles of recipient targe cells (Gong et al. 2019). Research has shown that the expression of miR-195, miR-24, miR-7-1-5p, and miR-223-3p is upregulated in serum derived exosomes of PD patients, while the expression of miR-19b is decreasing (Cao et al. 2017, Citterio et al. 2023). In the PD mouse model, miR-137 expression is increased in blood derived extracellular vesicles (Jiang et al. 2019).

Natural exosomes possess inherent advantages, making them ideal carriers for miRNA drugs (Izco et al. 2022). Firstly, exosomes can escape phagocytosis and low immune response (Li et al. 2021). Secondly, exosomes can fuse with the cellular membrane, enabling them to cross the blood-brain barrier (BBB), which is an important advantage in the treatment of central nervous system diseases (Dong 2018). However, there are important issues that warrant further exploration and discussion. The intricate internal structure of natural exosomes and the limited understanding of their role in disease and health processes make it challenging to leverage these exosomes for PD treatment (Kooijmans et al. 2012). Investigators still need to determine which types of cells are most effective in producing large quantities of extracellular vesicles with specific therapeutic applications (Colao et al. 2018, Ayala-Mar et al. 2019, Rankin-Turner et al. 2021). Meanwhile, it should be noted that exosomes sort miRNAs into exosomes through specific cargo-sorting machineries (Pastuzyn et al. 2018). Exosomes, once liberated into the extracellular milieu, embark on a complex journey, undergoing a cascade of biological events that culminate in their interaction with target cells. To exert their biological functions, these exosomes must fuse with the cell membrane, facilitating the release of their contents into the cytoplasm—a process termed endosomal escape. This critical step is essential for the exosomes' contents to exert their intended effects. Should the contents fail to achieve endosomal escape and remain confined within the endosomal pathway, they face a different fate. Trapped within this system, cargo may be targeted for lysosomal degradation, recycled inside cells, or expelled back into the extracellular environment. In such cases, the exosomes' contents lose biological activity and fail to modulate the functions of the target cells (Somiya 2020, O’Brien et al. 2022). Given the disadvantages of natural exosomes, an increasing number of studies aim to develop artificial exosomes based on biological hybridization techniques (Li et al. 2021).

Currently, one strategy involves engineering natural cell-derived exosomes to target specific cell types. In the second approach exploits the intrinsic characteristics of cell-derived exosomes to fabricate nanoscale drug delivery systems (Dommelen et al. 2012). Researchers used mesenchymal stem cells from the trophoblast stage derived from embryonic stem cells (T-MSCs) with uniform phenotypic profiles, which can stably produce their exosomes (T-MSCs-Exo) and have ability to cross the blood–brain barrier. The team further explored the molecular mechanisms involved in the protection of dopaminergic neurons in the PD model. T-MSCs-Exo may inhibit the expression level of the target gene NOX4 by delivering miR-100-5p, thereby reducing ROS production through the NOX4-ROS-Nrf2-axis, alleviating oxidative stress, and improving DA neuron damage in PD (He et al. 2023). Mutiple other studies have reported that in animal models treated with MPTP and cell models treated with MMP^+^, adipose-derived stem cell derived exosomes overexpression miR-188-3p restrain autophagy and cell apoptosis, suppress inflammatory response, ameliorate movement impairment, indicating the potential of miR-188-3p as a target for PD treatment (Li et al. 2021).

The nanoscale drug delivery system (NDDS) is fundamentally changing the treatment landscape of PD by providing innovative solutions to the challenges posed by traditional treatment methods (Baskin et al. 2021). These advanced systems utilize the power of nanotechnology to enhance drug bioavailability, ensuring that a larger proportion of administered drugs achieve expected goals within the CNS (Eleftheriadou et al. 2020). This is particularly important for PD, as the efficacy of treatments such as levodopa may be significantly affected by unpredictable absorption and widespread peripheral metabolism (Jankovic 2002). One of the most significant advantages of NDDS is their targeted drug delivery capacity. By altering the surface properties of nanoparticles, these systems can precisely target specific cells or tissues within the CNSc, such as dopaminergic neurons that are crucial for PD pathology. This targeted approach not only maximizes treatment efficacy, but also minimizes the risk of off-target effects, thereby reducing the likelihood of adverse side effects (Roco 2003). Current research focusing on NNDS drug therapy for PD, which also encompasses: (1) Strategies to overcome the BBB, enhancing more effective drug delivery to the brain (Saraiva et al. 2016). (2) These systems can regulate gene expression by delivering nucleic acids like siRNA or plasmid DNA, offering a potential disease-modifying approach for PD (Izco et al. 2019). To date, in PD treatment, despite the promising prospects of NDDS, few studies have described NNDS for miRNA delivery.

As our understanding of PD and nanotechnology continues to evolve, the role of NDDS in managing this complex disease will continue to expand. The potential of these systems to provide new and more effective treatment options for PD patients is enormous, bringing new hope to both patients and healthcare providers (Accomasso et al. 2018).

## Conclusion

MicroRNAs play critical roles in the pathogenesis of Parkinson’s disease by regulating various cellular processes involved in neurodegeneration. Dysregulation of miRNAs contributes to oxidative stress, mitochondrial dysfunction, neuroinflammation, and protein aggregation, all of which are hallmarks of PD. Understanding the specific roles of miRNAs in PD pathways provides potential therapeutic targets for intervention and opens up possibilities for the development of miRNA-based therapies. Further research is needed to elucidate the complex regulatory networks of miRNAs in PD and validate their potential as diagnostic and prognostic biomarkers.

## Data Availability

No datasets were generated or analysed during the current study.

## References

[CR1] Accomasso L, Cristallini C, Giachino C (2018) Risk assessment and risk minimization in nanomedicine: a need for predictive, alternative, and 3Rs strategies. Front Pharmacol 9:228. 10.3389/fphar.2018.00228 29662451 PMC5890110

[CR2] Angelopoulou E, Paudel YN, Piperi C (2019) miR-124 and Parkinson’s disease: A biomarker with therapeutic potential. Pharmacol Res 150:104515. 10.1016/j.phrs.2019.104515 31707035

[CR3] Anglicheau D, Muthukumar T, Suthanthiran M (2010) MicroRNAs: small RNAs with big effects. Transplantation 27(2):105–112. 10.1097/TP.0b013e3181e913c2 PMC3094098 20574417

[CR4] Armstrong MJ, Okun MS (2020) Diagnosis and Treatment of Parkinson Disease: A Review. JAMA 11(6):548–560. 10.1001/jama.2019.22360 32044947

[CR5] Asikainen S, Rudgalvyte M, Heikkinen L et al (2010) Global microRNA expression profiling of Caenorhabditis elegans Parkinson’s disease models. J Mol Neurosci 41(1):210–218. 10.1007/s12031-009-9325-1 20091141

[CR6] Ayala-Mar S, Donoso-Quezada J, Gallo-Villanueva RC, Perez-Gonzalez VH, Gonzalez-Valdez J (2019) Recent advances and challenges in the recovery and purification of cellular exosomes. Electrophoresis 40(23–24):3036–3049. 10.1002/elps.201800526 31373715 PMC6972601

[CR7] Baskin J, Jeon JE, Lewis SJG (2021) Nanoparticles for drug delivery in Parkinson’s disease. J Neurol 268(5):1981–1994. 10.1007/s00415-020-10291-x 33141248

[CR8] Bhattacharjee N, Borah A (2016) Oxidative stress and mitochondrial dysfunction are the underlying events of dopaminergic neurodegeneration in homocysteine rat model of Parkinson’s disease. Neurochem Int 101:48–55. 10.1016/j.neuint.2016.10.001 27732886

[CR9] Blauwendraat C, Nalls MA, Singleton AB (2020) The genetic architecture of Parkinson’s disease. Lancet Neurol 19(2):170–178. 10.1016/S1474-4422(19)30287-X 31521533 PMC8972299

[CR10] Bloem BR, Okun MS, Klein C (2021) Parkinson’s disease. Lancet 397(10291):2284–2303. 10.1016/S0140-6736(21)00218-X 33848468

[CR11] Bock FJ, Tait SWG (2020) Mitochondria as multifaceted regulators of cell death. Nat Rev Mol Cell Biol 21(2):85–100. 10.1038/s41580-019-0173-8 31636403

[CR12] Bodai L, Borosta R, Ferencz A, Kovacs M, Zsindely N (2024) The role of miR-137 in neurodegenerative disorders. Int J Mol Sci. 10.3390/ijms25137229 39000336 PMC11241563

[CR13] Calabresi P, Di Lazzaro G, Marino G, Campanelli F, Ghiglieri V (2023) Advances in understanding the function of alpha-synuclein: implications for Parkinson’s disease. Brain 1(9):3587–3597. 10.1093/brain/awad150 PMC10473562 37183455

[CR14] Caldi Gomes L, Galhoz A, Jain G et al (2022) Multi-omic landscaping of human midbrains identifies disease-relevant molecular targets and pathways in advanced-stage Parkinson’s disease. Clin Transl Med 12(1):e692. 10.1002/ctm2.692 35090094 PMC8797064

[CR15] Cao XY, Lu JM, Zhao ZQ et al (2017) MicroRNA biomarkers of Parkinson’s disease in serum exosome-like microvesicles. Neurosci Lett 644:94–99. 10.1016/j.neulet.2017.02.045 28223160

[CR16] Cardo LF, Coto E, Ribacoba R et al (2014) MiRNA profile in the substantia nigra of Parkinson’s disease and healthy subjects. J Mol Neurosci 54(4):830–836. 10.1007/s12031-014-0428-y 25284245

[CR17] Citterio LA, Mancuso R, Agostini S, Meloni M, Clerici M (2023) Serum and exosomal miR-7-1-5p and miR-223-3p as possible biomarkers for Parkinson’s disease. Biomolecules. 10.3390/biom13050865 37238734 PMC10216839

[CR18] Colao IL, Corteling R, Bracewell D, Wall I (2018) Manufacturing exosomes: a promising therapeutic platform. Trends Mol Med 24(3):242–256. 10.1016/j.molmed.2018.01.006 29449149

[CR19] Cressatti M, Juwara L, Galindez JM et al (2020) Salivary microR-153 and microR-223 Levels as Potential Diagnostic Biomarkers of Idiopathic Parkinson’s Disease. Mov Disord 35(3):468–477. 10.1002/mds.27935 31800144

[CR20] Cring MR, Sheffield VC (2022) Gene therapy and gene correction: targets, progress, and challenges for treating human diseases. Gene Ther 29(1–2):3–12. 10.1038/s41434-020-00197-8 33037407

[CR21] Dauer W, Przedborski S (2003) Parkinson’s Disease. Neuron 39(6):889–909. 10.1016/s0896-6273(03)00568-3 12971891

[CR22] De Bartolo MI, Belvisi D, Mancinelli R et al (2024) A systematic review of salivary biomarkers in Parkinson’s disease. Neural Regen Res 1(12):2613–2625. 10.4103/NRR.NRR-D-23-01677 PMC11168506 38595280

[CR23] Dehay B, Bourdenx M, Gorry P et al (2015) Targeting alpha-synuclein for treatment of Parkinson’s disease: mechanistic and therapeutic considerations. Lancet Neurol 14(8):855–866. 10.1016/S1474-4422(15)00006-X 26050140 PMC5217462

[CR24] Deverman BE, Ravina BM, Bankiewicz KS, Paul SM, Sah DWY (2018) Gene therapy for neurological disorders: progress and prospects. Nat Rev Drug Discov 17(10):767. 10.1038/nrd.2018.158 30206384

[CR25] Deverman BE, Ravina BM, Bankiewicz KS, Paul SM, Sah DWY (2018) Gene therapy for neurological disorders: progress and prospects. Nat Rev Drug Discov 17(9):641–659. 10.1038/nrd.2018.110 30093643

[CR26] Di Rita A, Maiorino T, Bruqi K, Volpicelli F, Bellenchi GC, Strappazzon F (2020) miR-218 inhibits mitochondrial clearance by targeting PRKN E3 ubiquitin ligase. Int J Mol Sci. 10.3390/ijms21010355 31948106 PMC6981953

[CR27] Diener C, Keller A, Meese E (2022) Emerging concepts of miRNA therapeutics: from cells to clinic. Trends Genet 38(6):613–626. 10.1016/j.tig.2022.02.006 35303998

[CR28] Dionisio PA, Amaral JD, Rodrigues CMP (2021) Oxidative stress and regulated cell death in Parkinson’s disease. Ageing Res Rev 67:101263. 10.1016/j.arr.2021.101263 33540042

[CR29] Dong X (2018) Current strategies for brain drug delivery. Theranostics 8(6):1481–1493. 10.7150/thno.21254 29556336 PMC5858162

[CR30] Doroszkiewicz J, Groblewska M, Mroczko B (2022) Molecular biomarkers and their implications for the early diagnosis of selected neurodegenerative diseases. Int J Mol Sci. 10.3390/ijms23094610 35563001 PMC9100918

[CR31] Dorsey ER, Bloem BR (2018) The Parkinson pandemic-a call to action. JAMA Neurol 75(1):9–10. 10.1001/jamaneurol.2017.3299 29131880

[CR32] Doxakis E (2010) Post-transcriptional Regulation of α-Synuclein Expression by mir-7 and mir-153. J Biol Chem 285(17):12726–34. 10.1074/jbc.M109.086827 20106983 PMC2857101

[CR33] Eldeeb MA, Thomas RA, Ragheb MA, Fallahi A, Fon EA (2022) Mitochondrial quality control in health and in Parkinson’s disease. Physiol Rev 1(4):1721–1755. 10.1152/physrev.00041.2021 35466694

[CR34] Eleftheriadou D, Kesidou D, Moura F, Felli E, Song W (2020) Redox-Responsive Nanobiomaterials-Based Therapeutics for Neurodegenerative Diseases. Small 16(43):e1907308. 10.1002/smll.201907308 32940007

[CR35] Elkouzi A, Vedam-Mai V, Eisinger RS, Okun MS (2019) Emerging therapies in Parkinson disease - repurposed drugs and new approaches. Nat Rev Neurol 15(4):204–223. 10.1038/s41582-019-0155-7 30867588 PMC7758837

[CR36] Esteves M, Cristovao AC, Vale A, Machado-Pereira M, Ferreira R, Bernardino L (2024) MicroRNA-124-3p Modulates Alpha-Synuclein Expression Levels in a Paraquat-Induced in vivo Model for Parkinson’s Disease. Neurochem Res 49(7):1677–1686. 10.1007/s11064-024-04130-y 38451434 PMC11144150

[CR37] Fan Z, Pan YT, Zhang ZY et al (2020) Systemic activation of NLRP3 inflammasome and plasma α-synuclein levels are correlated with motor severity and progression in Parkinson’s disease. J Neuroinflammation 17(1):11. 10.1186/s12974-019-1670-6 31915018 PMC6950934

[CR38] Farh KK, Grimson A, Jan C et al (2005) The widespread impact of mammalian MicroRNAs on mRNA repression and evolution. Science 16(5755):1817–1821. 10.1126/science.1121158 16308420

[CR39] Flusberg DA, Sorger PK (2015) Surviving apoptosis: life-death signaling in single cells. Trends Cell Biol 25(8):446–458. 10.1016/j.tcb.2015.03.003 25920803 PMC4570028

[CR40] Foltynie T, Bruno V, Fox S, Kuhn AA, Lindop F, Lees AJ (2024) Medical, surgical, and physical treatments for Parkinson’s disease. Lancet 20(10423):305–324. 10.1016/S0140-6736(23)01429-0 38245250

[CR41] Fricker M, Tolkovsky AM, Borutaite V, Coleman M, Brown GC (2018) Neuronal cell death. Physiol Rev 98(2):813–880. 10.1152/physrev.00011.2017 29488822 PMC5966715

[CR42] Gebert LFR, MacRae IJ (2019) Regulation of microRNA function in animals. Nat Rev Mol Cell Biol 20(1):21–37. 10.1038/s41580-018-0045-7 30108335 PMC6546304

[CR43] Gong C, Tian J, Wang Z et al (2019) Functional exosome-mediated co-delivery of doxorubicin and hydrophobically modified microRNA 159 for triple-negative breast cancer therapy. J Nanobiotechnol 3(1):93. 10.1186/s12951-019-0526-7 PMC6721253 31481080

[CR44] Gonzalez-Horta A (2015) The Interaction of Alpha-synuclein with Membranes and its Implication in Parkinson’s Disease: A Literature Review. Nat Prod Commun 10(10):1775–1778 26669123

[CR45] Gonzalez-Rodriguez P, Zampese E, Stout KA et al (2021) Disruption of mitochondrial complex I induces progressive parkinsonism. Nature 599(7886):650–656. 10.1038/s41586-021-04059-0 34732887 PMC9189968

[CR46] Graves SM, Xie Z, Stout KA et al (2020) Dopamine metabolism by a monoamine oxidase mitochondrial shuttle activates the electron transport chain. Nat Neurosci 23(1):15–20. 10.1038/s41593-019-0556-3 31844313 PMC7257994

[CR47] Grosso Jasutkar H, Oh SE, Mouradian MM (2022) Therapeutics in the Pipeline Targeting alpha-Synuclein for Parkinson’s Disease. Pharmacol Rev 74(1):207–237. 10.1124/pharmrev.120.000133 35017177 PMC11034868

[CR48] Han QQ, Le WD (2023) NLRP3 Inflammasome-Mediated Neuroinflammation and Related Mitochondrial Impairment in Parkinson’s Disease. Neurosci Bull 39(5):832–844. 10.1007/s12264-023-01023-y 36757612 PMC10169990

[CR49] Han R, Liu Y, Li S, Li XJ, Yang W (2023) PINK1-PRKN mediated mitophagy: differences between in vitro and in vivo models. Autophagy 19(5):1396–1405. 10.1080/15548627.2022.2139080 36282767 PMC10240983

[CR50] Haney MJ, Klyachko NL, Zhao Y et al (2015) Exosomes as drug delivery vehicles for Parkinson’s disease therapy. J Control Release 10:207:18–30. 10.1016/j.jconrel.2015.03.033 PMC4430381 25836593

[CR51] Haque ME, Akther M, Jakaria M, Kim IS, Azam S, Choi DK (2020) Targeting the microglial NLRP3 inflammasome and its role in Parkinson’s disease. Mov Disord 35(1):20–33. 10.1002/mds.27874 31680318

[CR52] He L, Hannon GJ (2004) MicroRNAs: small RNAs with a big role in gene regulation. Nat Rev Genet 5(7):522–531. 10.1038/nrg1379 15211354

[CR53] He S, Wang Q, Chen L, He YJ, Wang X, Qu S (2023) miR-100a-5p-enriched exosomes derived from mesenchymal stem cells enhance the anti-oxidant effect in a Parkinson’s disease model via regulation of Nox4/ROS/Nrf2 signaling. J Transl Med 24(1):747. 10.1186/s12967-023-04638-x PMC10594913 37875930

[CR54] Hebert SS, De Strooper B (2007) Molecular biology. miRNAs in neurodegeneration. Science 31(5842):1179–1180. 10.1126/science.1148530 17761871

[CR55] Henrich MT, Oertel WH, Surmeier DJ, Geibl FF (2023) Mitochondrial dysfunction in Parkinson’s disease - a key disease hallmark with therapeutic potential. Mol Neurodegener 11(1):83. 10.1186/s13024-023-00676-7 PMC10640762 37951933

[CR56] Hill DR, Huters AD, Towne TB et al (2023) Parkinson’s Disease: Advances in Treatment and the Syntheses of Various Classes of Pharmaceutical Drug Substances. Chem Rev 13(23):13693–13712. 10.1021/acs.chemrev.3c00479 37975808

[CR57] Hinderer C, Katz N, Buza EL et al (2018) Severe Toxicity in Nonhuman Primates and Piglets Following High-Dose Intravenous Administration of an Adeno-Associated Virus Vector Expressing Human SMN. Hum Gene Ther 29(3):285–298. 10.1089/hum.2018.015 29378426 PMC5865262

[CR58] Hu Q, Wang G (2016) Mitochondrial dysfunction in Parkinson’s disease. Transl Neurodegener 5:14. 10.1186/s40035-016-0060-6 27453777 PMC4957882

[CR59] Huang S, Chen Z, Fan B et al (2021) A selective NLRP3 inflammasome inhibitor attenuates behavioral deficits and neuroinflammation in a mouse model of Parkinson’s disease. J Neuroimmunol 354:577543. 10.1016/j.jneuroim.2021.577543 33714750

[CR60] Huenchuguala S, Segura-Aguilar J (2024) Single-neuron neurodegeneration as a degenerative model for Parkinson’s disease. Neural Regen Res 19(3):529–535. 10.4103/1673-5374.380878 37721280 PMC10581573

[CR61] Izco M, Blesa J, Schleef M et al (2019) Systemic Exosomal Delivery of shRNA Minicircles Prevents Parkinsonian Pathology. Mol Ther 4(12):2111–2122. 10.1016/j.ymthe.2019.08.010 PMC6904801 31501034

[CR62] Izco M, Carlos E, Alvarez-Erviti L (2022) The Two Faces of Exosomes in Parkinson’s Disease: From Pathology to Therapy. Neuroscientist 28(2):180–193. 10.1177/1073858421990001 33530851

[CR63] Jackson AL, Bartz SR, Schelter J et al (2003) Expression profiling reveals off-target gene regulation by RNAi. Nat Biotechnol 21(6):635–637. 10.1038/nbt831 12754523

[CR64] Jackson AL, Linsley PS (2010) Recognizing and avoiding siRNA off-target effects for target identification and therapeutic application. Nat Rev Drug Discov 9(1):57–67. 10.1038/nrd3010 20043028

[CR65] Jankovic J (2002) Levodopa strengths and weaknesses. Neurology 58(4 Suppl 1):S19-32. 10.1212/wnl.58.suppl_1.s19 11909982

[CR66] Jankovic J, Tan EK (2020) Parkinson’s disease: etiopathogenesis and treatment. J Neurol Neurosurg Psychiatry 91(8):795–808. 10.1136/jnnp-2019-322338 32576618

[CR67] Jia X, Wang F, Han Y et al (2016) miR-137 and miR-491 Negatively Regulate Dopamine Transporter Expression and Function in Neural Cells. Neurosci Bull 32(6):512–522. 10.1007/s12264-016-0061-6 27628529 PMC5563834

[CR68] Jiang Y, Chen J, Sun Y et al (2021) Profiling of Differentially Expressed MicroRNAs in Saliva of Parkinson’s Disease Patients. Front Neurol 12:738530. 10.3389/fneur.2021.738530 34899562 PMC8660675

[CR69] Jiang Y, Liu J, Chen L et al (2019) Serum secreted miR-137-containing exosomes affects oxidative stress of neurons by regulating OXR1 in Parkinson’s disease. Brain Res 1722:146331. 10.1016/j.brainres.2019.146331 31301273

[CR70] Jomova K, Raptova R, Alomar SY et al (2023) Reactive oxygen species, toxicity, oxidative stress, and antioxidants: chronic diseases and aging. Arch Toxicol 97(10):2499–2574. 10.1007/s00204-023-03562-9 37597078 PMC10475008

[CR71] Kayagaki N, Webster JD, Newton K (2024) Control of cell death in health and disease. Annu Rev Pathol 19:157–180. 10.1146/annurev-pathmechdis-051022-014433 37788577

[CR72] Keiser MS, Ranum PT, Yrigollen CM et al (2021) Toxicity after AAV delivery of RNAi expression constructs into nonhuman primate brain. Nat Med 27(11):1982–1989. 10.1038/s41591-021-01522-3 34663988 PMC8605996

[CR73] Kern F, Fehlmann T, Violich I et al (2021) Deep sequencing of sncRNAs reveals hallmarks and regulatory modules of the transcriptome during Parkinson’s disease progression. Nat Aging 1(3):309–322. 10.1038/s43587-021-00042-6 37118411

[CR74] Kim J, Fiesel FC, Belmonte KC et al (2016) miR-27a and miR-27b regulate autophagic clearance of damaged mitochondria by targeting PTEN-induced putative kinase 1 (PINK1). Mol Neurodegener 11(1):55. 10.1186/s13024-016-0121-4 27456084 PMC4960690

[CR75] Kim J, Inoue K, Ishii J et al (2007) A MicroRNA feedback circuit in midbrain dopamine neurons. Science 31(5842):1220–1224. 10.1126/science.1140481 PMC2782470 17761882

[CR76] Kojima R, Bojar D, Rizzi G et al (2018) Designer exosomes produced by implanted cells intracerebrally deliver therapeutic cargo for Parkinson’s disease treatment. Nat Commun 3(1):1305. 10.1038/s41467-018-03733-8 PMC5880805 29610454

[CR77] Kooijmans SA, Vader P, van Dommelen SM, van Solinge WW, Schiffelers RM (2012) Exosome mimetics: a novel class of drug delivery systems. Int J Nanomed 7:1525–1541. 10.2147/IJN.S29661 PMC3356169 22619510

[CR78] Kuo MC, Liu SC, Hsu YF, Wu RM (2021) The role of noncoding RNAs in Parkinson’s disease: biomarkers and associations with pathogenic pathways. J Biomed Sci 18(1):78. 10.1186/s12929-021-00775-x PMC8603508 34794432

[CR79] Lai X, Eberhardt M, Schmitz U, Vera J (2019) Systems biology-based investigation of cooperating microRNAs as monotherapy or adjuvant therapy in cancer. Nucleic Acids Res 5(15):7753–7766. 10.1093/nar/gkz638 PMC6735922 31340025

[CR80] Langston JW (2017) The MPTP Story. J Parkinsons Dis 7(s1):S11–S19. 10.3233/JPD-179006 28282815 PMC5345642

[CR81] Lee E, Hwang I, Park S et al (2019) MPTP-driven NLRP3 inflammasome activation in microglia plays a central role in dopaminergic neurodegeneration. Cell Death Differ 26(2):213–228. 10.1038/s41418-018-0124-5 29786072 PMC6329843

[CR82] Lee RC, Feinbaum RL, Ambros V (1993) The *C. elegans* heterochronic gene *lin-4* encodes small RNAs with antisense complementarity to *lin-14*. Cell 75(5):843–54. 10.1016/0092-8674(93)90529-y 8252621

[CR83] Li D, Mastaglia FL, Fletcher S, Wilton SD (2020) Progress in the molecular pathogenesis and nucleic acid therapeutics for Parkinson’s disease in the precision medicine era. Med Res Rev 40(6):2650–2681. 10.1002/med.21718 32767426 PMC7589267

[CR84] Li D, Yang H, Ma J, Luo S, Chen S, Gu Q (2018) MicroRNA-30e regulates neuroinflammation in MPTP model of Parkinson’s disease by targeting Nlrp3. Hum Cell 31(2):106–115. 10.1007/s13577-017-0187-5 29274035 PMC5852205

[CR85] Li J, Yang D, Li Z et al (2023) PINK1/Parkin-mediated mitophagy in neurodegenerative diseases. Ageing Res Rev 84:101817. 10.1016/j.arr.2022.101817 36503124

[CR86] Li Q, Wang Z, Xing H, Wang Y, Guo Y (2021) Exosomes derived from miR-188-3p-modified adipose-derived mesenchymal stem cells protect Parkinson’s disease. Mol Ther Nucleic Acids 5:23:1334–1344. 10.1016/j.omtn.2021.01.022 PMC7920810 33717653

[CR87] Li YJ, Wu JY, Liu J et al (2021) Artificial exosomes for translational nanomedicine. J Nanobiotechnol 19(1):242. 10.1186/s12951-021-00986-2 PMC8359033 34384440

[CR88] Malpartida AB, Williamson M, Narendra DP, Wade-Martins R, Ryan BJ (2021) Mitochondrial Dysfunction and Mitophagy in Parkinson’s Disease: From Mechanism to Therapy. Trends Biochem Sci 46(4):329–343. 10.1016/j.tibs.2020.11.007 33323315

[CR89] Masato A, Plotegher N, Boassa D, Bubacco L (2019) Impaired dopamine metabolism in Parkinson’s disease pathogenesis. Mol Neurodegener 20(1):35. 10.1186/s13024-019-0332-6 PMC6728988 31488222

[CR90] Mendell JR, Al-Zaidy SA, Rodino-Klapac LR et al (2021) Current Clinical Applications of In Vivo Gene Therapy with AAVs. Mol Ther 3(2):464–488. 10.1016/j.ymthe.2020.12.007 PMC7854298 33309881

[CR91] Meredith GE, Rademacher DJ (2011) MPTP mouse models of Parkinson’s disease: an update. J Parkinsons Dis 1(1):19–33. 10.3233/JPD-2011-11023 23275799 PMC3530193

[CR92] Morris HR, Spillantini MG, Sue CM, Williams-Gray CH (2024) The pathogenesis of Parkinson’s disease. Lancet 20(10423):293–304. 10.1016/S0140-6736(23)01478-2 38245249

[CR93] Moujalled D, Strasser A, Liddell JR (2021) Molecular mechanisms of cell death in neurological diseases. Cell Death Differ 28(7):2029–2044. 10.1038/s41418-021-00814-y 34099897 PMC8257776

[CR94] Mouradian MM (2012) MicroRNAs in Parkinson’s disease. Neurobiol Dis 46(2):279–84. 10.1016/j.nbd.2011.12.046 22245218

[CR95] Nakatogawa H (2020) Mechanisms governing autophagosome biogenesis. Nat Rev Mol Cell Biol 21(8):439–458. 10.1038/s41580-020-0241-0 32372019

[CR96] Napoletano F, Baron O, Vandenabeele P, Mollereau B, Fanto M (2019) Intersections between regulated cell death and autophagy. Trends Cell Biol 29(4):323–338. 10.1016/j.tcb.2018.12.007 30665736

[CR97] Nelson PT, Wilfred BR (2009) In situ hybridization is a necessary experimental complement to microRNA (miRNA) expression profiling in the human brain. Neurosci Lett 4(2):69–72. 10.1016/j.neulet.2009.04.044 PMC3171000 19393719

[CR98] Ng J, Barral S, Waddington SN, Kurian MA (2023) Gene Therapy for Dopamine Dyshomeostasis: From Parkinson’s to Primary Neurotransmitter Diseases. Mov Disord 38(6):924–936. 10.1002/mds.29416 37147851 PMC10946997

[CR99] Nies YH, Mohamad Najib NH, Lim WL, Kamaruzzaman MA, Yahaya MF, Teoh SL (2021) MicroRNA Dysregulation in Parkinson’s Disease: A Narrative Review. Front Neurosci 15:660379. 10.3389/fnins.2021.660379 33994934 PMC8121453

[CR100] O’Brien J, Hayder H, Zayed Y, Peng C (2018) Overview of microRNA biogenesis, mechanisms of actions, and circulation. Front Endocrinol (Lausanne) 9:402. 10.3389/fendo.2018.00402 30123182 PMC6085463

[CR101] O’Brien K, Ughetto S, Mahjoum S, Nair AV, Breakefield XO (2022) Uptake, functionality, and re-release of extracellular vesicle-encapsulated cargo. Cell Rep 39(2):110651. 10.1016/j.celrep.2022.110651 35417683 PMC9074118

[CR102] Pagano G, Taylor KI, Anzures Cabrera J et al (2024) Prasinezumab slows motor progression in rapidly progressing early-stage Parkinson’s disease. Nat Med 30(4):1096–1103. 10.1038/s41591-024-02886-y 38622249 PMC11031390

[CR103] Pastuzyn ED, Day CE, Kearns RB et al (2018) The neuronal gene Arc encodes a repurposed retrotransposon Gag protein that mediates intercellular RNA transfer. Cell 172(1–2):275-288 e18. 10.1016/j.cell.2017.12.024 29328916 PMC5884693

[CR104] Perinan MT, Brolin K, Bandres-Ciga S et al (2022) Effect Modification between Genes and Environment and Parkinson’s Disease Risk. Ann Neurol 92(5):715–724. 10.1002/ana.26467 35913124 PMC9588606

[CR105] Pfeifer GP (2024) DNA damage and Parkinson’s disease. Int J Mol Sci. 10.3390/ijms25084187 38673772 PMC11050701

[CR106] Rankin-Turner S, Vader P, O’Driscoll L, Giebel B, Heaney LM, Davies OG (2021) A call for the standardised reporting of factors affecting the exogenous loading of extracellular vesicles with therapeutic cargos. Adv Drug Deliv Rev 173:479–491. 10.1016/j.addr.2021.04.012 33862168 PMC8191593

[CR107] Ravanidis S, Bougea A, Papagiannakis N et al (2020) Validation of differentially expressed brain-enriched microRNAs in the plasma of PD patients. Ann Clin Transl Neurol 7(9):1594–1607. 10.1002/acn3.51146 32860338 PMC7480914

[CR108] Roco MC (2003) Nanotechnology: convergence with modern biology and medicine. Curr Opin Biotechnol 14(3):337–346. 10.1016/s0958-1669(03)00068-5 12849790

[CR109] Saadh MJ, Faisal A, Adil M et al (2024) Parkinson’s disease and microRNAs: a duel between inhibition and stimulation of apoptosis in neuronal cells. Mol Neurobiol. 10.1007/s12035-024-04111-w 38520611

[CR110] Sadlon A, Takousis P, Alexopoulos P, Evangelou E, Prokopenko I, Perneczky R (2019) MiRNAs identify shared pathways in Alzheimer’s and Parkinson’s diseases. Trends Mol Med 25(8):662–672. 10.1016/j.molmed.2019.05.006 31221572

[CR111] Saraiva C, Esteves M, Bernardino L (2017) MicroRNA: basic concepts and implications for regeneration and repair of neurodegenerative diseases. Biochem Pharmacol 141:118–131. 10.1016/j.bcp.2017.07.008 28709951

[CR112] Saraiva C, Praca C, Ferreira R, Santos T, Ferreira L, Bernardino L (2016) Nanoparticle-mediated brain drug delivery: Overcoming blood-brain barrier to treat neurodegenerative diseases. J Control Release 235:34–47. 10.1016/j.jconrel.2016.05.044 27208862

[CR113] Sayed D, Abdellatif M (2011) MicroRNAs in development and disease. Physiol Rev 91(3):827–887. 10.1152/physrev.00006.2010 21742789

[CR114] Schildknecht S, Di Monte DA, Pape R, Tieu K, Leist M (2017) Tipping Points and Endogenous Determinants of Nigrostriatal Degeneration by MPTP. Trends Pharmacol Sci 38(6):541–555. 10.1016/j.tips.2017.03.010 28442167

[CR115] Segal M, Slack FJ (2020) Challenges identifying efficacious miRNA therapeutics for cancer. Expert Opin Drug Discov 15(9):987–992. 10.1080/17460441.2020.1765770 32421364 PMC7415578

[CR116] Slyk Z, Stachowiak N, Malecki M (2024) Recombinant adeno-associated virus vectors for gene therapy of the central nervous system: delivery routes and clinical aspects. Biomedicines. 10.3390/biomedicines12071523 39062095 PMC11274884

[CR117] Somiya M (2020) Where does the cargo go?: solutions to provide experimental support for the “extracellular vesicle cargo transfer hypothesis.” J Cell Commun Signal 14(2):135–146. 10.1007/s12079-020-00552-9 32060725 PMC7272534

[CR118] Starhof C, Hejl AM, Heegaard NHH et al (2019) The biomarker potential of cell-free microRNA from cerebrospinal fluid in Parkinsonian Syndromes. Mov Disord 34(2):246–254. 10.1002/mds.27542 30557454

[CR119] Stein CS, McLendon JM, Witmer NH, Boudreau RL (2022) Modulation of miR-181 influences dopaminergic neuronal degeneration in a mouse model of Parkinson’s disease. Mol Ther Nucleic Acids 14:1–15. 10.1016/j.omtn.2022.02.007 PMC8899134 35280925

[CR120] Su Y, Deng MF, Xiong W et al (2019) MicroRNA-26a/Death-Associated Protein Kinase 1 Signaling Induces Synucleinopathy and Dopaminergic Neuron Degeneration in Parkinson’s Disease. Biol Psychiatry 85(9):769–781. 10.1016/j.biopsych.2018.12.008 30718039 PMC8861874

[CR121] Sun Q, Wang S, Chen J et al (2019) MicroRNA-190 alleviates neuronal damage and inhibits neuroinflammation via Nlrp3 in MPTP-induced Parkinson’s disease mouse model. J Cell Physiol 234(12):23379–23387. 10.1002/jcp.28907 31232472

[CR122] Tansey MG, Wallings RL, Houser MC, Herrick MK, Keating CE, Joers V (2022) Inflammation and immune dysfunction in Parkinson disease. Nat Rev Immunol 22(11):657–673. 10.1038/s41577-022-00684-6 35246670 PMC8895080

[CR123] Teresak P, Lapao A, Subic N, Boya P, Elazar Z, Simonsen A (2022) Regulation of PRKN-independent mitophagy. Autophagy 18(1):24–39. 10.1080/15548627.2021.1888244 33570005 PMC8865282

[CR124] Thome AD, Harms AS, Volpicelli-Daley LA, Standaert DG (2016) microRNA-155 Regulates Alpha-Synuclein-Induced Inflammatory Responses in Models of Parkinson Disease. J Neurosci 24(8):2383–2390. 10.1523/JNEUROSCI.3900-15.2016 PMC4764660 26911687

[CR125] Tryphena KP, Anuradha U, Kumar R et al (2023) Understanding the Involvement of microRNAs in Mitochondrial Dysfunction and Their Role as Potential Biomarkers and Therapeutic Targets in Parkinson’s Disease. J Alzheimers Dis 94(s1):S187–S202. 10.3233/JAD-220449 35848027 PMC10473154

[CR126] van Dommelen SM, Vader P, Lakhal S et al (2012) Microvesicles and exosomes: opportunities for cell-derived membrane vesicles in drug delivery. J Control Release 20(2):635–644. 10.1016/j.jconrel.2011.11.021 22138068

[CR127] Vargas JNS, Hamasaki M, Kawabata T, Youle RJ, Yoshimori T (2023) The mechanisms and roles of selective autophagy in mammals. Nat Rev Mol Cell Biol 24(3):167–185. 10.1038/s41580-022-00542-2 36302887

[CR128] Vellingiri B, Chandrasekhar M, Sri Sabari S et al (2022) Neurotoxicity of pesticides - A link to neurodegeneration. Ecotoxicol Environ Saf 243:113972. 10.1016/j.ecoenv.2022.113972 36029574

[CR129] Vitale I, Pietrocola F, Guilbaud E et al (2023) Apoptotic cell death in disease-Current understanding of the NCCD 2023. Cell Death Differ 30(5):1097–1154. 10.1038/s41418-023-01153-w 37100955 PMC10130819

[CR130] Voet S, Srinivasan S, Lamkanfi M, van Loo G (2019) Inflammasomes in neuroinflammatory and neurodegenerative diseases. Embo Mol Med 11(6):e10248. 10.15252/emmm.201810248 31015277 PMC6554670

[CR131] Wang Q, Liu Y, Zhou J (2015) Neuroinflammation in Parkinson’s disease and its potential as therapeutic target. Transl Neurodegener 4:19. 10.1186/s40035-015-0042-0 26464797 PMC4603346

[CR132] Wang X, Winter D, Ashrafi G et al (2011) PINK1 and Parkin target Miro for phosphorylation and degradation to arrest mitochondrial motility. Cell 11(4):893–906. 10.1016/j.cell.2011.10.018 PMC3261796 22078885

[CR133] Watanabe H, Dijkstra JM, Nagatsu T (2024) Parkinson’s disease: cells succumbing to lifelong dopamine-related oxidative stress and other bioenergetic challenges. Int J Mol Sci. 10.3390/ijms25042009 38396687 PMC10888576

[CR134] Yang Y, Li Y, Yang H, Guo J, Li N (2021) Circulating MicroRNAs and Long Non-coding RNAs as Potential Diagnostic Biomarkers for Parkinson’s Disease. Front Mol Neurosci 14:631553. 10.3389/fnmol.2021.631553 33762908 PMC7982809

[CR135] Yao L, Ye Y, Mao H et al (2018) MicroRNA-124 regulates the expression of MEKK3 in the inflammatory pathogenesis of Parkinson’s disease. J Neuroinflammation 12(1):13. 10.1186/s12974-018-1053-4 PMC5767033 29329581

[CR136] Yao L, Zhu Z, Wu J et al (2019) MicroRNA-124 regulates the expression of p62/p38 and promotes autophagy in the inflammatory pathogenesis of Parkinson’s disease. FASEB J 33(7):8648–8665. 10.1096/fj.201900363R 30995872

[CR137] Yoo M, Choi DC, Murphy A, Ahsan AM, Junn E (2023) MicroRNA-593-5p contributes to cell death following exposure to 1-methyl-4-phenylpyridinium by targeting PTEN-induced putative kinase 1. J Biol Chem 299(5):104709. 10.1016/j.jbc.2023.104709 37060996 PMC10196868

[CR138] Yuan J, Ofengeim D (2024) A guide to cell death pathways. Nat Rev Mol Cell Biol 25(5):379–395. 10.1038/s41580-023-00689-6 38110635

[CR139] Zhang D, Zhang J, Wang Y et al (2023) Targeting epigenetic modifications in Parkinson’s disease therapy. Med Res Rev 43(5):1748–1777. 10.1002/med.21962 37119043

[CR140] Zhang J, Zhao M, Yan R et al (2021) MicroRNA-7 Protects Against Neurodegeneration Induced by alpha-Synuclein Preformed Fibrils in the Mouse Brain. Neurotherapeutics 18(4):2529–2540. 10.1007/s13311-021-01130-6 34697773 PMC8804150

[CR141] Zhang S, Cheng Z, Wang Y, Han T (2021) The Risks of miRNA Therapeutics: In a Drug Target Perspective. Drug Des Devel Ther 15:721–733. 10.2147/DDDT.S288859 33654378 PMC7910153

[CR142] Zhang X, Xu A, Lv J et al (2020) Development of small molecule inhibitors targeting NLRP3 inflammasome pathway for inflammatory diseases. Eur J Med Chem 185:111822. 10.1016/j.ejmech.2019.111822 31699536

[CR143] Zhou J, Zhao Y, Li Z et al (2020) miR-103a-3p regulates mitophagy in Parkinson’s disease through Parkin/Ambra1 signaling. Pharmacol Res 160:105197. 10.1016/j.phrs.2020.105197 32942015

[CR144] Zhou Y, Lu M, Du RH et al (2016) MicroRNA-7 targets Nod-like receptor protein 3 inflammasome to modulate neuroinflammation in the pathogenesis of Parkinson’s disease. Mol Neurodegener 11(1):28. 10.1186/s13024-016-0094-3 27084336 PMC4833896

